# Study on the method of enucleation of anterior uterine fibroids by transverse incision of the lower uterine segment during cesarean section

**DOI:** 10.1186/s12884-021-04226-1

**Published:** 2021-11-03

**Authors:** Yan Dai, Li Xia, Jinxiao Lin, Rongli Xu, Wenqiang You

**Affiliations:** Department of obstetrics, Fujian Maternity and Child Health Hospital, Affiliated Hospital of Fujian Medical University, FuZhou, 350001 FuJian China

**Keywords:** Cesarean section, Uterine myomectomy, Uterine large fibroids, Prognosis

## Abstract

**Introduction:**

A retrospective study was conducted to investigate the effectiveness and feasibility of fibroid enucleation in the anterior wall of the uterus by transverse uterine incision during cesarean section.

**Methods:**

The medical history, surgical data, preoperative and postoperative changes in the blood system, and complications of 90 pregnant women who underwent myomectomy of the anterior uterine wall during cesarean section at the second Department of Maternal and Child Health Hospital of Fujian Province were analyzed retrospectively.

**Results:**

No significant differences were noted in the leiomyoma number, pathological type, preoperative and postoperative hemoglobin level, perioperative bleeding incidence, blood transfusion frequency, postoperative fever incidence, and duration of lochia between the study and control groups. The proportion of large fibroids was slightly higher in the study group than in the control group (*p* < 0.05), and the operation time and average hospitalization time were slightly longer in the study group than in the control group (*p* < 0.05). The distribution of type III–V fibroids was slightly more in the study group than in the control group (*p* < 0.05), and the distribution of type VI fibroids in the study group was less than that in the control group (p < 0.05).

**Conclusion:**

Fibroid enucleation is safe and effective in the anterior wall of the uterus through the lower uterine transverse incision in cesarean section. It has the potential to reduce the risk of pelvic and intrauterine adhesions in the future.

## Introduction

Uterine fibroids are common benign tumors in women of childbearing age, causing increased menstrual volume, pelvic pain, fibroid degeneration, and infertility [1]. Most cases may present with no obvious clinical symptoms, and many women are diagnosed with uterine fibroids using obstetric ultrasound after pregnancy [2]. According to statistics, the incidence of uterine fibroids during pregnancy ranging from 1.6 to 10.7%, which may result in miscarriage, premature birth, abnormal fetal position, placental abruption, obstructed birth passage, postpartum hemorrhage, and other obstetric complications [2]. Compared with pregnancy combined small fibroids, pregnancy combined with large fibroids of the uterus is more prone to obstetric complications, often requiring cesarean section to terminate the pregnancy [3]. It is still controversial whether it is necessary to remove uterine fibroids at the same time during cesarean section. Some scholars believe that uterine blood flow is rich during pregnancy, and enucleation of fibroids at this time is likely to cause uncontrollable bleeding [4]. In addition to pedicled uterine fibroids, enucleation of fibroids at the same time during cesarean section is not recommended [2]. However, with the progress of surgical hemostasis to prevent postpartum hemorrhage, many clinical studies have shown that cesarean section while eviscerating uterine fibroids can be a safe operation [5–7], for some patients can save the time and cost borne by reoperation [8].

But in fact, the additional incision was required for the removal of single or multiple fibroids during cesarean section, and adhesion formation is a matter of fact in myomectomies [9]. In order to minimize abdominal and intrauterine adhesions as possible, the present study introduces a surgical technigue, which can remove anterior uterine wall myoma by caesarean section incision without additional uterine incision. The aim of the present investigation was to evaluate the safety and effectiveness of the innovative method in patients with anterior wall uterine segment fibroids.

## Method

This study is a retrospective analysis of pregnant women diagnosed with anterior uterine fibroids and admitted to the second Department of Obstetrics, Fujian maternal and child health hospital, from January 2015 to December 2019

### Inclusion criteria

Hysteromyoma enucleation performed simultaneously during cesarean section, which was performed by the corresponding author of this study. Intraoperative examination showed that the myoma was located in the anterior wall of the uterus (excluding types 0, I, and VII), the diameter of the myoma was ≥3 cm, and the postoperative pathology confirmed that the myoma was a uterine leiomyoma. This study included 90 patients who were divided into two groups based on the method of uterine fibroid enucleation: 50 patients with anterior uterine fibroids enucleated by an incision through the serous layer (control group) and 40 patients with anterior uterine fibroids enucleated by an incision through the lower segment of the uterus (study group). The following data were obtained from the medical records: maternal age, number of pregnancies and births, age of gestation, weight, height, body mass index (BMI), pregnancy complications, neonatal weight, Apgar score, indications, and type of cesarean section (emergency or elective), myoma type using the International Federation of Obstetrics and Gynecology (FIGO) uterine leiomyoma type 9 classification method [1], divided into three groups: type 0–II submucous type, III–V type intermuscular type. VI–VII is subserous type, and type VIII is other special types or sites of leiomyoma, cervical leiomyoma), the maximum diameter of leiomyoma (measured by a pathologist, divided into 3–5 cm and ≥ 5 cm), location of leiomyoma (recording the distance from the lower edge of leiomyoma to uterine incision, divided into ≤2 cm, 2–5 cm, ≥5 cm), number of leiomyomas (single or multiple), operation time (in minutes, from skin incision to skin closure), intraoperative blood loss (data from surgical and anesthetic surgery reports), blood transfusion, methods of myoma enucleation (myoma enucleation through incision margin or serosa of the uterus), hemostatic measures used in the process of myoma enucleation (such as strong oxytocin, parauterine vascular ligation, and uterine compression suture), preoperative and postoperative hemoglobin and hematocrit levels, the main complications, postoperative hospital stay, lochia 42 days after delivery, uterine involution and reexamination of pelvic color ultrasound. This study was approved by the Ethics Committee of Fujian Maternal and Child Health Hospital, and all patients provided written informed consent.

### Methods of enucleation of uterine leiomyoma

**Fig. 1:**
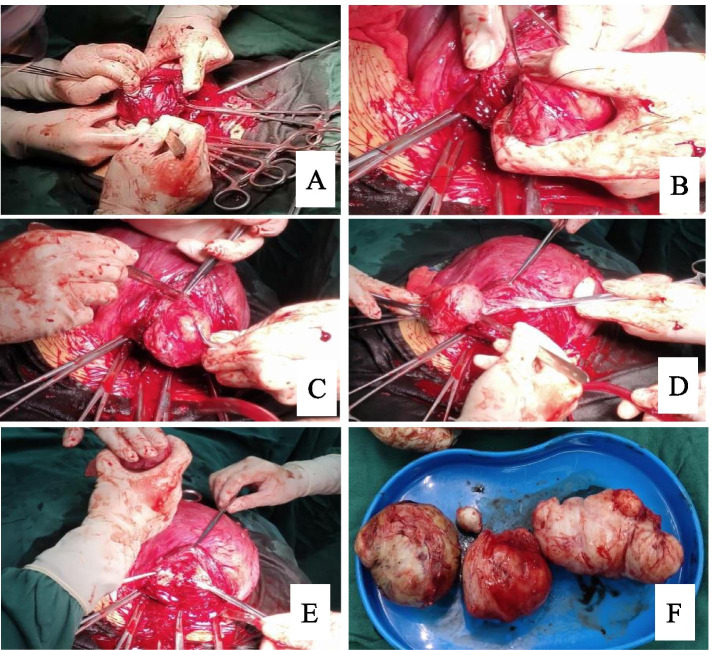
Enucleation of hysteromyoma through the upper edge of cesarean section incision. **A**. Cut to the tumor wall at the most protruding part of the incisal margin; **B**. The root tissue of the tumor is sutured with 1–0 vicryl in a seperated manner; **C**-**E**. Enucleation of hysteromyoma; **F**. All myomas of the anterior wall of uterus were enucleated

**Fig. 2:**
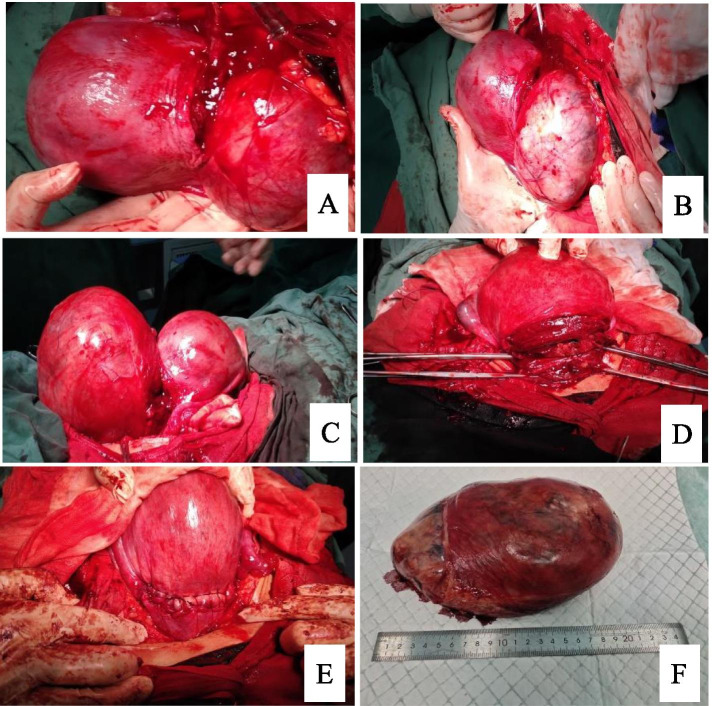
Enucleation of hysteromyoma through the lower edge of cesarean section incision. **A**-**C**. Large fibroid of anterior wall of lower margin of cesarean section; **D**. Enucleation of large fibroid in the anterior wall of the lower uterine segment through the lower edge of cesarean section incision; **E**. Suture the caesarean section of the uterine incision; **F**. Enucleated large fibroid of uterus

After administration of spinal epidural anesthesia, the patient is placed in the supine position, and the Pfannenstiel incision for routine cesarean section is performed. Then, the fetus and placenta are delivered, the uterine cavity is wiped with a wet gauze, and the uterine incision to stop the bleeding. Next, 100 μG cabetoxin is intravenously administered to promote uterine contraction. If poor uterine contraction is observed, parauterine vascular ligation is performed, and a strong contractile agent injection (such as Carprost aminobuttriol injection) is used before enucleation of uterine fibroids. The uterus is then held outside the abdominal incision to detect the myoma number, location, and size immediately. In the study group, the hysteromyoma is cut and enucleated through the lower incision edge of the uterus, which is palpated to confirm the position of the hysteromyoma. Next, the distance from the lower edge of the hysteromyoma to the incision edge is measured for the hysteromyoma above the incision, and the distance from the upper edge of the hysteromyoma to the incision edge is measured for the hysteromyoma below the incision. The fundus uteri is held with the left hand, and the assistant helps in applying pressure to the myoma from the serosa to myometrium to the endometrium (Fig. 1a), so that the myoma moves in the incision direction until the incision edge swells. According to the depth of the myoma, it is cut to the tumor wall at the most swelled part of the incision edge, the tumor is clamped with cloth towel pliers, it is pulled outward until the tumor nucleus is completely exposed, the root is clamped with curved pliers, and the root tissue of the tumor is sutured with 1–0 vicryl in a seperated manner, then the leiomyoma is removed while tightening the root. The tumor cavity is sutured and closed until the incision is made. If no bleeding is noted, a cesarean section incision is routinely sutured. In the control group, the uterine incision is sutured first, and the myoma is then enucleated by a traditional subserosal incision. The tumor cavity is sutured intermittently with 1–0 vicryl. Finally, the uterine seromuscular layer is sutured continuously (Figs. 1 and 2).

### Statistical analysis

SPSS software (IBM version 21.0) was used for statistical analysis. Continuous variables are presented as mean ± standard deviation or median (minimum-maximum), and categorical variables are presented as numbers or percentages where appropriate. One-way analysis of variance, the Kruskal–Wallis test, and Pearson’s chi-square test were used for comparisons. Differences were considered statistically significant at two-tailed *p* values of < 0.05.

## Results

### Clinical characteristics of the subjects

The study included 90 pregnant women, with an average age of 34.7 ± 4.58 years. The patients were divided into two groups according to the method of enucleation of the largest leiomyoma: in 40 patients (44%), enucleation was performed through the incision margin of the lower segment of the uterus (study group), and in 50 patients (56%), enucleation was performed through a subserous incision (control group). There were no significant differences in age, BMI, weight, height, gestational age, gravidity, parity, previous history of cesarean section or myomectomy, primipara, emergency cesarean section, abnormal fetal position, and neonatal weight between the study and control groups. Among the 90 patients, no significant difference was noted in the distribution of obstetrical complications (such as gestational diabetes mellitus, gestational hypertension, premature rupture of membranes, placenta previa, and placental abruption) between the two groups (all *p* > 0.05). Among the 90 patients, there were five cases of fetal growth restriction (6%), six cases of fetal distress (7%), one case of fetal death at 22 weeks of pregnancy, and no cases of neonatal asphyxia. The clinical and sociological data of the two groups are presented in Table 1.

**Table 1:** Patients’ clinical and demographic data

Characteristics	Total	Study group	Control group	*p*-value
Patient (n)	90	40	50	
Age (years)	34.7 ± 4.58	34.58 ± 4.25	34.80 ± 4.87	0.82
BMI (Kg/m2)	26.76 ± 3.25	26.66 ± 3.59	26.84 ± 2.99	0.79
Body weight (Kg)	67.72 ± 7.89	67.20 ± 7.77	68.15 ± 8.04	0.57
Height(cm)	159.21 ± 4.87	159.01 ± 4.30	159.38 ± 5.31	0.72
Gestational age (weeks)	37.9(22.5–41)	37.74(22.5–41)	38.03(29.5–41)	0.93
Gravidity (times)	2.4(1–7)	2.4(1–5)	2.5(1–7)	0.81
Parity (times)	0.7(0–3)	0.6(0–3)	0.8(0–2)	0.13
Previous cesarean section (n [%])	33[37]	12[30]	21[42]	0.24
Previous myomectomy (n [%])	3[3]	2[5]	1[2]	0.43
Primipara (n[%])	38[42]	21[53]	17[34]	0.08
Emergency cesarean section (n [%])	21[23]	11[27]	10[20]	0.40
Abnormal fetal position (n[%])	12[13]	8[20]	4[8]	0.10
Neonatal weight (g)	3160.5(550–4570)	3108.98(550–4570)	3201.70(1030–4535)	0.54
Gestational diabetes mellitus (n [%])	22[24]	9[23]	13[26]	0.70
Hypertension complicating pregnancy (n [%])	9[10]	5[13]	4[8]	0.48
Premature rupture of membranes (n[%])	11[12]	5[13]	6[12]	0.94
Placenta previa (n[%])	5[6]	2[5]	3[6]	0.78
Placenta abruption (n[%])	1[1]	1[3]	0[0]	0.91
Fetal distress (n[%])	6[7]	1[3]	5[10]	0.16
Stillbirth (n[%])	1[1]	1[3]	0[0]	0.44
Fetal growth restriction (n[%])	5[6]	1[3]	4[8]	0.26
Neonatal asphyxia (n[%])	0	0	0	

### Comparison of the uterine leiomyoma characteristics between the two groups

There was a significant difference in the size of the largest fibroids between the study and control groups. The number of cases with the largest fibroid diameter of ≥5 cm was higher in the study group than in the control group (73% vs. 42% *p* = 0.00). In terms of the distance between the largest fibroids and the incision edge of the lower segment of the uterus, the number of cases in the study group was higher than that in the control group at a distance of ≤2 cm (53% vs. 6%, *p* = 0.00), whereas in terms of the distance of ≥5 cm, the number of cases in the control group was higher than that in the study group (46% vs. 0%, p = 0.00). There was no significant difference in the distance of 2–5 cm between the study and control groups (48% vs. 48%, *p* = 0.96). According to the comparison of the distribution of fibroids between the two groups according to the FIGO classification, there was no significant difference in type II fibroids between the two groups (3% vs. 2%, *p* = 0.58), whereas the proportion of type III–V fibroids was higher in the study group than in the control group (80% vs. 60%, *p* = 0.04) and the proportion of type VI fibroids was lesser in the study group than in the control group (18% vs. 38%, *p* = 0.03). There was no significant difference in the number of fibroids (single or multiple) and the type of pathological diagnosis between the study and control groups (all *p* > 0.05). Comparison of the characteristics of uterine fibroids between the two groups is shown in Table 2.

**Table 2:** Characteristics of uterine fibroids data

Characteristics	Total	Study group	Control group	p-value
**Patient (n)**	90	40	50	
**Number of leiomyoma**				
Solitary myoma(n[%])	44[49]	18[45]	26[52]	0.51
Multiple myoma (≥2)(n[%])	46[51]	22[55]	24[48]	0.51
**The size of the largest myoma**	6.2(3–20)	7.2(3–20)	5.3(3–14)	0.01
Size of leiomyoma < 5 cm (n[%])	40[44]	11[28]	29[58]	0.00
Size of leiomyoma ≥5 cm (n[%])	50[56]	29[73]	21[42]	0.00
**FIGO classification of leiomyoma**				
Type II(n[%])	2[2]	1[3]	1[2]	0.58
Type III-V(n[%])	62[69]	32[80]	30[60]	0.04
Type VI(n[%])	26[29]	7[18]	19[38]	0.03
**The distance between the myoma and the incisal margin**				
≤2 cm(n[%])	24[27]	21[53]	3[6]	0.00
2-5 cm(n[%])	43[48]	19[48]	24[48]	0.96
≥5 cm(n[%])	23[26]	0[0]	23[46]	0.00
**Pathological diagnosis of leiomyoma**				
No denaturation (n[%])	42[47]	21[53]	21[42]	0.32
Red degeneration (n[%])	39[43]	16[40]	23[46]	0.57
Cystic degeneration (n[%])	0	0	0	0
Glass transformation (n[%])	9[10]	3[8]	6[12]	0.72

### Comparison of the changes in operation and blood-related indexes

Among the 90 patients, except for the indication for obstetrical cesarean section, 14 patients (15.6%) underwent cesarean section simply because of uterine fibroids. Uterine fibroids were used as an indication of cesarean section surgery, and no significant difference was noted in the distribution between the two groups (*p* = 0.10). The total operation time in the study group was 40–162 min (median 83.3 min), which is slightly longer than the 42–137 min (median 72.5 min) in the control group, and the difference was statistically significant (*p* = 0.04). The postoperative hospital stay in the study group was slightly longer than that in the control group (median 3.6 vs. 3.2, *p* = 0.01). There was no significant difference in the hemoglobin and hematocrit levels and hemoglobin and hematocrit changes between the two groups before and after surgery (all *p* > 0.05). Of the 90 patients, one patient had postpartum hemorrhage (1.1%), three had requests for blood transfusion (3.3%), and five had fever after surgery (5.6%). There was no significant difference between the two groups (all p > 0.05) in the postpartum hemorrhage and transfusion and fever. There were no operative complications in either group. All patients were followed up for more than half a year, and there was no difference in the time of lochia cleaning between the two groups. A comparison of the operation and blood-related indexes between the two groups is shown in Table 3.

**Table 3:** Data about surgical procedures and hematological analysis

Characteristics	Total	Study group	Control group	p-value
Patient (n)	90	40	50	
Uterine leiomyoma(n[%])	14[15.6]	9[22.5]	5[10.0]	0.10
Total duration of operation(min)	77.3(40–162)	83.3(40–162)	72.5(42–137)	0.04
Preoperative hemoglobin(g/L)	118.8 ± 12.1	118.9 ± 10.9	118.7 ± 13.2	0.94
Postoperative hemoglobin(g/L)	102.9 ± 15.1	100.9 ± 14.9	104.5 ± 15.1	0.17
Hemoglobin change	16.0(−1–59)	18.05(−1–56)	14.28(0–59)	0.06
Preoperative hematocrit(%)	35.2 ± 3.1	35.17 ± 2.75	35.15 ± 3.42	0.97
Postoperative hematocrit(%)	30.7 ± 4.1	30.12 ± 3.96	31.2 ± 4.3	0.12
Hematocrit changes	4.7(−8.6–17.2)	5.1(−8.6–16)	3.9(−2.4–17.2)	0.07
Intraoperative bleeding(ml)	486.7(300–1500)	477.5(300–800)	494(300–1500)	0.80
Postpartum hemorrhage(n[%])	1[1.1]	0[0]	1[2.0]	0.91
Blood transfusion(n[%])	3[3.3]	1[2.5]	2[4.0]	0.70
Postoperative fever(n[%])	5[5.6]	2[5.0]	3[6.0]	0.84
Postoperative hospital stay (days)	3.4(3–6.5)	3.6(3–6.5)	3.2(3–5)	0.01
Operative complications(n[%])	0	0	0	
Duration of lochia (days)	30(10–90)	29(14–90)	30(10–90)	0.86

## Discussion

The incidence of uterine fibroids increases with age, especially between 30 and 40 years of age [10]. With an increase in the childbearing age, the incidence of uterine leiomyoma in pregnancy increases accordingly, and complications, such as early pregnancy bleeding and abortion, premature delivery, premature rupture of membranes, and placental abruption, may occur. However, in most cases, patients do not present with any clinical symptoms, and many women are even present with uterine leiomyoma during obstetrical ultrasound examinations after pregnancy [11]. In terms of pregnancy with uterine leiomyoma without obstruction of the birth canal or trial delivery without contraindications, most pregnant women can still be encouraged to opt for a vaginal delivery. However, if the myoma is located in the lower segment of the uterus, it will often lead to obstruction of the birth canal and abnormal fetal position, which is an indication of cesarean section. For example, 15.6% patients in this study chose cesarean section because of simple large uterine fibroids.

Whether or not to remove uterine fibroids simultaneously during cesarean section remains controversial. According to the traditional view, uterine hyperemia during pregnancy is obvious, and myoma is generally enlarged under the influence of progesterone [12]. Uterine myomectomy may result in perioperative complications, such as massive bleeding, prolonged operation time, and postoperative infection. In severe cases, there is even a risk of hysterectomy. Therefore, except for subserous myoma with a pedicle, performing hysteromyomectomy at the same time as cesarean section is not recommended [2, 4, 13]. However, in recent years, with the development of hemostatic technology for cesarean section, an increasing number of studies have shown that hysteromyoma enucleation performed simultaneously during the cesarean section is safe and feasible [6, 7, 14–16]. In this study, 90 patients who underwent cesarean section with myomectomy of the anterior uterine wall were selected; 40 patients had uterine myomas that were eviscerated through the edge of the uterine incision in the study group, and the remaining 50 patients had uterine leiomyomas treated with the traditional serous layer enucleation in the control group. There were no surgical complications in all patients in this study. The results showed that hysteromyoma enucleation performed simultaneously during the cesarean section is safe.

It is crucial to make a good evaluation and countermeasure before the operation meticulously. Evaluation items included whether the selected cases were appropriate, whether the surgeons were experienced, complete rescue equipment, adequate blood sources, etc. Usually this procedure is done in a tertiary hospital. The same medical team performed all the operations in this study, and the chief surgeon had 15–25 years of surgical experience; the patient’s tolerance and willingness were thoroughly evaluated before the operation. In this study, there were three cases of blood transfusion. One patient in the study group had postpartum hemorrhage caused by multiple myoma evisceration, and two patients in the control group had complications of severe anemia before surgery; therefore, they were treated with blood transfusion before surgery. Myomectomy is feasible in a tertiary medical center, which equipped with adequate manpower and blood products [8].

Uterine myoma evisceration is performed simultaneously during cesarean section, especially in cases of large fibroids, and according to the traditional subserous myomectomy method, it often aggravates the wound on the surface of the uterus, thus increasing the risk of pelvic adhesion in the future. Hatirnaz et al. proposed a method to remove the uterine leiomyoma from the endometrium to solve this problem [12]. They believed that the endometrium, myometrium, and serosal layer could be healed smoothly through an endometrial incision to enucleate uterine fibroids and directly merged to reduce the risk of pelvic adhesions in the future [9]. Although endometrial myomectomy helps in avoiding the formation of adhesions between the serosa and the surrounding organs, adhesions may occur in the uterine cavity [12]. Our team innovatively adopted the method of uterine fibroid enucleation from the edge of the uterine incision to solve this problem. This method is suitable for myomas of the anterior wall of the middle and lower segments of the uterus. Without adding a new uterine incision, the myoma of the uterus can be removed, which can preserve the integrity of the uterine tissue as much as possible and reduce the possibility of uterine and pelvic adhesion in the future. In this study, there were no significant differences in the preoperative and postoperative hemoglobin levels, perioperative bleeding incidence, blood transfusion frequency, postoperative fever incidence, and complications between the study and control groups. Therefore, this study proved that the innovative application of the method of removing myoma from the anterior wall of the uterus through the edge of the uterine incision is a safe and feasible method compared with the traditional method. In addition, the myoma of the uterus can be removed without increasing uterine wounds, which can reduce the possibility of uterine and pelvic adhesion in the future. For obstetricians, there is one more option to remove uterine fibroids simultaneously during cesarean section, especially for young patients with reproductive requirements, thereby reducing the incision on the uterine surface and uterine cavity, which is conducive to postoperative recovery.

Performing myomectomy simultaneously after the cesarean section has its own advantages and disadvantages. Previous studies have shown that simultaneous enucleation of uterine fibroids by cesarean section may increase the length of surgery and hospital stay, but this can reduce the future morbidity of patients due to multiple surgeries, anesthesia complications, and out-of-pocket costs [8, 12, 15, 17, 18]. As this study said, the operation time and average hospitalization stay were longer in the study group, which may be related to the higher proportion of major myomas (diameter ≥ 5 cm) in the study group than that in the control group. Compared with small leiomyomas, large myomas take more time to eviscerate, repair, and recover after surgery.

The method of cutting into the edge of the uterine incision and enucleating the uterine leiomyoma is more suitable for myomas of the anterior wall of the middle and lower segment of the uterus, especially for large leiomyomas. Research showed that the myoma pseudocapsule in a pregnant uterus was larger than that of a non-pregnant uterus and myometrium was more elastic and less delicateduring pregnancy, so fibroids could be easily excised [12]. On the other hand, the anterior uterine wall muscle layer is occupied by large fibroids, and the muscularis becomes thin, so the fibroids are more likely to be pushed to the incision margin of cesarean section. It helps the operator easily to remove the tumor nucleus from the edge of the incision. Myomas close to the fundus and corner uterine segment are far from the incision edge of the cesarean section, and be extruded to cesarean section incision edge difficultly, therefore, endometrial enucleation of uterine fibroids has no obvious advantage. Usually, if the lower margin of the uterine fibroid is more than 5 cm from the lateral margin of the incision, we use the method of serosal fibroid enucleation or endometrial enucleation instead. In this study, the proportion of type III–V fibroids as per the FIGO classification of leiomyomas in the study group was slightly high, and the proportion of the lower edge of leiomyomas with the uterine incision edge of ≤2 cm was high, which reflected the applicability of this method.

Pregnancy with uterine leiomyoma increases the difficulty of obstetric management, and a large myoma in the lower segment of the uterus often requires a cesarean section in terms of terminating the pregnancy because the myoma obstructs the birth canal. Many pregnant women prefer to eviscerate the myoma of the lower part of the uterus simultaneously during cesarean section, which can reduce the possibility of postpartum hemorrhage and secondary operation caused by uterine weakness caused by myoma. Therefore, this study displayed the full use of the transverse incision of the lower segment of the uterus to remove the myoma of the intermuscular type of the anterior wall of the middle and lower segment of the uterus, which cannot aggravate the wound surface of the uterus and reduce the risk of pelvic and uterine adhesion in the future. This method is easy to perform and is choice for hysteromyoma evisceration during cesarean section.

This study had some limitations. The number of patients was limited, and the follow-up time was not enough. In addition, the impact on the long-term outcomes needs to be further reviewed by the prospective case-control studies.

## Data Availability

All data generated or analysed during this study are included in this published article.
